# Cadaveric Anatomical Study of Sural Nerve: Where is The Safe Area for Endoscopic Gastrocnemius Recession?

**DOI:** 10.2174/1874325001711011094

**Published:** 2017-09-30

**Authors:** Alvin Chin Kwong Tan, Zhi Hao Tang, Muhammad Farhan Bin Mohd Fadil

**Affiliations:** 1 Department of Orthopaedic Surgery, Khoo Teck Puat Hospital, 90 Yishun, Central Singapore, 768828, Singapore; 2 Department of Orthopaedic Surgery, Tan Tock Seng Hospital, 11 Jalan Tan Tock Seng Singapore, 308433, Singapore

**Keywords:** Gastrocnemius recession, Sural nerve, Calcaneal tuberosity, Gastrocnemius tendon, Achilles tendon

## Abstract

**Purpose:**

To ascertain in cadavers where the sural nerve crosses the gastro-soleus complex and where the gastrocnemius tendon merges with the Achilles tendon in relation to the calcaneal tuberosities.

**Methods:**

Twelve cadaveric lower limbs (6 right and 6 left) were dissected. The distances between the calcaneal tuberosities and the lateral border of the Achilles tendon where the sural nerve crosses from medial to lateral, as well as to the gastrocnemius tendon insertion into the Achilles tendon, were measured.

**Results:**

The mean and median longitudinal distances from the calcaneal tuberosity to where the sural nerve crosses the lateral border of the Achilles tendon are 9.9cm and 10cm respectively (range 7cm to 14cm). The mean and median longitudinal distances from the calcaneal tuberosity to where the gastrocnemius tendon inserts into the Achilles tendon are 19.9cm and 18.5cm (range 17cm to 25cm) respectively.

**Conclusion:**

It is generally safe to place the posterolateral incision more than 14cm above the calcaneal tuberosity to avoid the sural nerve if surgeons plan to use a posterolateral incision for endoscopic recession. The distance between the calcaneal tuberosity to the gastrocnemius tendon insertion into the Achilles tendon is too highly variable to be used as a landmark for locating the gastrocnemius insertion.

## 1 INTRODUCTION

The gastrocnemius recession was originally described to treat isolated gastrocnemius contractures in the paediatric population with neuromuscular disorders [1]. This procedure is now also being performed for gastrocnemius contractures in adults with conditions such as symptomatic hallux valgus, pes planus and plantar fasciitis [2-4].

Both open and endoscopic techniques can be used for gastrocnemius recession [5]. The Strayer [1] and Baumann [6] open procedures describe gastrocnemius fascia recession at different levels. The incision for performing the Strayer procedure is made 2cm distal to the gastrocnemius indentation, and the gastrocnemius fascia is sectioned 1 to 2 fingerbreadths distal to the musculotendinous junction. The incision for the Baumann procedure is over the proximal medial calf, where the deep surface of the gastrocnemius muscle is sectioned. The Vulpius procedure is a gastrocnemius-soleus recession done for plantarflexion contractures with both the gastrocnemius fascia and the soleus muscle sectioned [7].

But while the open gastrocnemius recession techniques have been the traditional gold standard, endoscopic techniques have gained popularity with its purported advantages of a smaller incision, better cosmesis and faster recovery [8]. Performing an endoscopic gastrocnemius recession involves placing a posteromedial and/or posterolateral portal over the calf 12cm to 14 cm above the calcaneal tuberosity. After the development of tissue planes, a cannula is advanced; through which an endoscope is introduced. The tendon is then transected under direct vision of the endoscope.

Owing to the limited endoscopic visualization, iatrogenic injuries to the sural nerve is one of the potential surgical complications [9]. The reported rate of sural nerve injuries ranges from 2% (open) to 16% (endoscopic)[10].

Our study aims to ascertain where the sural nerve crosses the gastro-soleus complex and where the gastrocnemius tendon merges with the Achilles tendon in relation to the calcaneal tuberosities. Better understanding of the anatomy of the sural nerve in relation to the gastro-soleus complex can decrease the risk of the injury.

## 2 MATERIALS AND METHODS

12 fresh frozen cadaveric adult human lower limbs were used for this study. The gastrocnemius origin from the posterior femoral condyle, as well as the knee and ankle joints were intact for each of the dissected specimens.

**Fig. (1):**
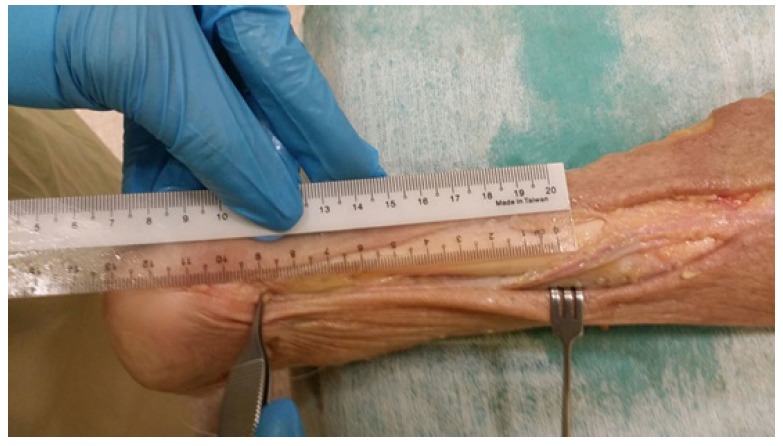
Measurement from the calcaneal tuberosity to where the sural nerve crosses the lateral border of the Achilles tendon.

**Fig. (2):**
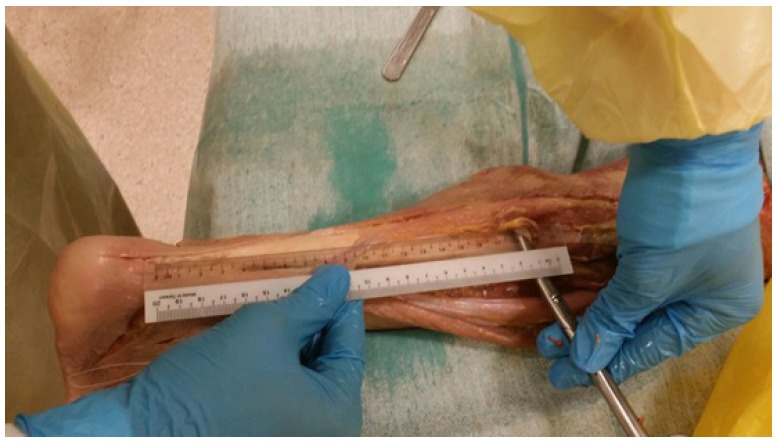
Measurement from the calcaneal tuberosity to point of insertion of the gastrocnemius tendon into the Achilles tendon.

Dissection and measurement of the cadaveric limbs were carried out by a single, fellowship trained Orthopaedic Surgeon. The limbs were placed in a prone position. A generous midline longitudinal incision was made from the middle of the popliteal fossa to the calcaneal tuberosity for each limb. The gastrocnemius muscles, gastrocnemius tendons and the insertion of the Achilles tendons into the calcaneal tuberosity were visualized. The sural nerve was also identified, explored and its course visualized for all 12 legs. The distances from the calcaneal tuberosity to where the sural nerve crosses the lateral border of the Achilles tendon (Fig. **1**) as well as the distances from the calcaneal tuberosity to where the gastrocnemius tendon inserts into the Achilles tendon (Fig. **2**) were recorded.

## 3 RESULTS

12 lower limb specimens (6 left and 6 right) were dissected and examined. There were 6 limbs from male cadavers, 1 limb from a female cadaver and 5 limbs from cadavers of unknown gender. As isolated lower limb specimens were used for this study, other anthropometric data were not available to us. The mean and median longitudinal distances from the calcaneal tuberosity to where the sural nerve crosses the lateral border of the Achilles tendon are 9.9cm and 10 cm, respectively (range 7 to 14cm). The mean and median longitudinal distances from the calcaneal tuberosity to where the gastrocnemius tendon inserts into the Achilles tendon are 19.9cm and 18.5cm (range 17 to 25cm), respectively.

## 4 DISCUSSION

The gastrocnemius originates from the posterior aspect of the femur, crossing the knee joint to insert into the into the calcaneal tuberosity *via* the Achilles tendon. The soleus muscle does not cross the knee joint as it arises from the posterior aspect of the tibia, the fibula and the interosseous membrane. It also inserts into the calcaneal tuberosity *via* the Achilles tendon.

This differential origin of the muscles forms the basis for the Silfverskiold test, which specifically measures gastrocnemius contracture by alternately relaxing and incorporating the muscle because of its origin proximal to the knee joint [11]. Keeping the subtalar joint neutral with the knee being extended, the ankle joint is passively dorsiflexed. The amount of ankle dorsiflexion is noted. The knee is then flexed with the ankle still passively dorsiflexed with the subtalar joint neutral. Any improvement in ankle dorsiflexion is then recorded. Any improvement in the ankle dorsiflexion when the knee is flexed compared to that when the knee is extended, is attributed to the tight gastrocnemius as flexing the knee relaxes the gastrocnemius, rather than the triceps surae complex.

The sural nerve is a sensory nerve formed by branches of the tibial nerve and the common peroneal nerve. Commonly, these are the medial sural cutaneous nerve and the peroneal communicating nerve; however variations in its formation exist. The nerve then runs down the posterior lateral aspect of the gastro-soleus complex and subsequently, the lateral border of the Achilles tendon. It supplies the posterior-lateal aspect of the leg, the lateral foot and the fifth toe [9] Injury to the sural nerve can result in sensory disturbances to the territory it supplies and a painful neuroma.

**Fig. (3):**
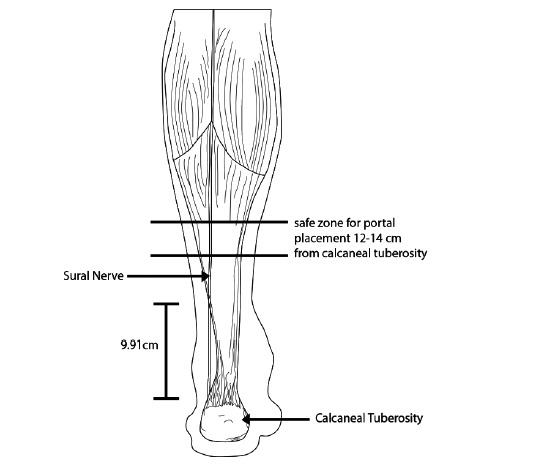
Safe zone for endoscopic portal placement.

Based on our results, the sural nerve crosses the lateral border of the Achilles tendon at a point no more than 14 cm proximal to the calcaneal tuberosity, with an average distance of 9.9cm (Fig. **3**). This correlates well with other previous studying looking at the anatomy of the sural nerve [9]. Thus, it is generally safe to place posterolateral incisions at least 14cm proximal to the calcaneal tuberosity if a posterolateral incision is planned for an endoscopic recession. However, injuries to the sural nerve during the gastrocnemius recession is still possible as it is lies posterior to the Achilles tendon and gastrocnemius tendon proximal to where it crosses the lateral border (Table **1**).

Our results also showed that the distance in which the gastrocnemius tendon insertions into the Achilles tendon is highly variable, ranging from 17 to 25cm. Based on our cadaveric study, using the calcaneal tuberosity as a guide to where the gastrocnemius tendon inserts into the Achilles tendon may not be reliable. A study by Pinney *et al.* [12] on patients who underwent the Strayer procedure through a posteromedial incision showed that the junction ranged from 10.7 to 20.9cm from the calcaneal tuberosity, with an average of 15.9cm. The ratio of this distance to the length of the leg was also variable from 0.33 to 0.6. It has been suggested that this junction is usually 18mm below the gastrocnemius indentation in this study and this is recommended as a landmark for making the posteromedial incision 2cm distal to the indentation and dissecting proximally.

## CONCLUSION

The strength of our study is that, it is based on human cadaveric specimens, which gives good representation of the location of the sural nerve that could be injured intra-operatively in actual practice. Nonetheless, it is limited by small number of cadaveric specimens.

## Figures and Tables

**Table 1:** Measurements of Sural Nerve and Gastrocnemius Tendon.

Cadaver	Laterality	Distance from calcaneal tuberosity to the sural nerve where it crosses the lateral border of Achilles tendon	Distance from calcaneal tuberosity to the insertion of gastrocnemius tendon into Achilles tendon
1 2 3 4 5 6 7 8 9 10 11 12	Right Left Right Right Right Right Left Left Left Left Left Right	13cm 8cm 9cm 8cm 11cm 10cm 10cm 14cm 10cm 7cm 9cm 10cm	25cm 18cm 17cm 18cm 18cm 17cm 19cm 22cm 18cm 22cm 25cm 20cm
Mean		9.9cm	19.9cm
Median		10cm	18.5cm
